# Modern contraceptive use among migrant and non-migrant women in Kenya

**DOI:** 10.1186/s12978-016-0183-3

**Published:** 2016-06-01

**Authors:** Rhoune Ochako, Ian Askew, Jerry Okal, John Oucho, Marleen Temmerman

**Affiliations:** Faculty of Medicine and Health Sciences, Ghent University, Ghent, Belgium; World Health Organization, Geneva, Switzerland; Population Council, P.O. Box 17643, 00100, Nairobi, Kenya; Population Studies and Research Institute, University of Nairobi, P.O. Box 30197-00100, Nairobi, Kenya; International Centre for Reproductive Health, Ghent University, Ghent, Belgium

**Keywords:** Kenya, Migration, Migration streams, Modern contraceptive use

## Abstract

**Background:**

Manifest socio-economic differences are a trigger for internal migration in many sub-Saharan settings including Kenya. An interplay of the social, political and economic factors often lead to internal migration. Internal migration potentially has significant consequences on an individual’s economic growth and on access to health services, however, there has been little research on these dynamics. In Kenya, where regional differentials in population growth and poverty reduction continue to be priorities in the post MDG development agenda, understanding the relationships between contraceptive use and internal migration is highly relevant.

**Methods:**

Using data from the 2008–09 Kenya Demographic and Health Survey (DHS), we analyze data from 5,905 women aged 15–49 years who reported being sexually active in the last 12 months prior to the survey. Bivariate and multivariate logistic regressions are fitted to predict correlates of contraceptive use in the presence of migration streams among other explanatory variables.

**Results:**

Modern contraceptive use was significantly higher among women in all migration streams (non-migrant urban (OR = 2.8, *p* < 0.001), urban-urban (OR = 2.0, *p* < 0.001), urban-rural (OR = 2.0, *p* < 0.001), rural-urban (OR = 2.6, *p* < 0.001), rural-rural (OR = 1.7, *p* < 0.001), than non-migrant rural women.

**Conclusion:**

Women who internally migrate within Kenya, whether from rural to urban or between urban centres, were more likely to use modern contraception than non-migrant rural women. This phenomenon appears to be due to selection, adaption and disruption effects which are likely to promote use of modern contraceptives. Programmatically, the differentials in modern contraceptive use by the different migration streams should be considered when designing family planning programmes among migrant and non-migrant women.

## Background

Internal migration plays an important role in explaining the population dynamics which consequently influence the population structure and distribution [1, 2]. Despite this important role, internal migration receives low priority by policy makers and governments in Kenya and other sub-Saharan African countries, in part due to knowledge gaps on the extent, nature and magnitude of internal migration and its nexus to health and overall well-being [2]. When people migrate, they interact with new social, cultural and economic contexts which potentially change their way of thinking and behavior to resemble that of the host community. While rural-rural migration remains the most predominant form of migration in Kenya, it is rural-urban migration that potentially brings change in the lives of migrants by offering knowledge, socio-economic opportunities and overall improved living standards [3].

In the recent past, there has been a shift from focusing on movement patterns for males to feminization of migration and the occurrence of other forms of migration streams [4]. The current attention on female migration and its associated health outcomes call for a particular understanding of the sexual and reproductive health needs of migrant females [4–7]. Contraceptive use among migrants therefore remains of interest to demographers, population scientists and policy makers due to its influence on fertility, sexual and reproductive health and the implications for provision of appropriate services [7, 8].

Migration can be a life changing event with profound consequences for sexual and reproductive health [9]. Migration from rural to urban areas is likely to increase access to contraception thereby increasing knowledge and uptake of sexual and reproductive health services [10]. Existing studies have shown that rural-urban migrants have lower fertility than non-migrants remaining in rural areas but have higher fertility than non-migrant urban residents [11–13]. According to the 2008–09 Kenya Demographic Health Survey (KDHS), married women in urban areas were more likely to use a contraceptive (53 %) than their rural counterparts (43 %). Additionally, the use of modern methods was generally higher in urban (47 %) than in rural areas (37 %) [14]. Total fertility rate also dropped from 4.9 children per women to 4.6 children per woman between 2003 and 2008–09 respectively. This decline was observed more among women in urban areas where there was a decline from 3.3 to 2.9 children per woman compared to a drop from 5.4 to 5.2 children per woman during the same period for rural women [14].

### Theoretical framework

In demographic literature, three theories: selection, adaption and disruption, are used to explain the causes of differentials between migrant and non-migrant women which in turn may explain the changes in their observed behaviour [15, 16]. The selection theory explains migrants as a self-selected group with characteristics different from non-migrants in rural areas due to their higher levels of education, later age at marriage, lower pre-migration fertility and participation in gainful employment [17]. These factors have been shown to have an effect before and after a migration event. The disruption theory, on the other hand, suggests that migration leads to physical separation of sexual partners which in turn helps postpone or space child bearing. The adaption theory proposes that socio-cultural norms in the migration destination will influence those moving from rural to urban areas. It is worth noting that these theories do not act in isolation given the dynamic relationship between them, hence the need to identify the effects of each to understand their implications on modern contraceptive use [18].

The Kenya Demographic and Health Survey (KDHS), conducted every five years since 1989 and the nationwide census have consistently provided comparable data on contraceptive use and consequently fertility changes. In the late 1980’s the country began a fertility transition; fertility decline was observed across all age groups and mainly attributed to improvements in child survival and use of modern contraceptives which helped achieve smaller desired family sizes [19]. For instance, use of modern contraceptives increased from 5.9 % (Kenya Fertility Survey) in 1970s to 31.5 % in 1998 (KDHS 1998). Additionally, the proportion of married women reporting no desire for more children increased from 17 to 53 % in the same period. The fertility transition witnessed in the 1980s and 1990s stalled and reversed from 2000 onwards. The stall was attributed to an increase in child mortality due to HIV and AIDS and shortages in contraceptives following diversion of resources from family planning programs to HIV prevention [20].

This paper focuses on the 2008–09 Kenya DHS as it involves data collected after these changes, including the stall in fertility decline witnessed in Kenya. We seek to explore modern contraceptive use among migrant and non-migrant women through bivariate and multivariate logistic models. We seek to answer the question of whether change in residential status has an influence on modern contraceptive use. Findings from this paper will be shared with the stakeholders working on programmes that seek to influence contraceptive use specifically among populations in mobility.

## Methods

### Source of data

This paper uses data from the 2008–09 Kenya Demographic and Health Survey (DHS) which is a nationally representative survey of women aged 15–49 years. From the 9,057 households interviewed, 8,767 women were found to be eligible and 8,444 were interviewed, giving a response rate of 96 %. The data were weighted to adjust for differences in probability of selection and non-response. As of March 2015, this was the latest survey data available for Kenya. This analysis is restricted to the 5,905 (weighted) women who reported being sexually active in the last 12 months prior to the survey; we excluded from this analysis any woman who reported they were pregnant at the time of the survey, regardless of the pregnancy duration and any woman who reported that they were infecund or sterile as they were not exposed to the risk of pregnancy.

### Study variables

The outcome variable, modern contraceptive use, was coded as a binary outcome into ‘*ye*s’ for women who reported using a modern method of contraception and ‘*no’* for women not using any method or those using folkloric and traditional methods of contraceptives. Contraceptive methods considered ‘modern’ included the pill, IUD, injectables, condom, female sterilization, male sterilization, norplant, lactational amenorrhea and female condom as classified by the DHS program. The key explanatory variable is migration stream, coded as a six-level variable as follows: non-migrant urban, non-migrant rural, urban-urban migrants, urban-rural migrants, rural-urban migrants and rural-rural migrants. The migration variable is generated using reports on current place of residence and previous place of residence as reported by the respondents. The DHS asked the question, “how long have you been living continuously in this (current) place of residence?” Those who answered ‘always’ were classified as non-migrants (either rural or urban), while those who answered in terms of ‘number of years lived at the current place of residence’ were further asked a question on previous place of residence before current residence to which they answered by stating previous residence as ‘city or in a town or in the countryside’. This information was further used to construct six migration streams namely: urban non-migrants, rural non-migrants, rural-urban, rural-rural, urban-urban, urban-rural. The inclusion of explanatory variables is informed by a conceptual framework that proposes the influence of socio-demographic factors (age of the woman, marital status, number of living children, religion, fertility preference, region of residence, and marital duration), and socio-economic factors (level of education, wealth index, occupation and hearing family planning message on media) on modern contraceptive use and migration stream.

### Data analysis

Analysis of the data was carried out using STATA v.14, descriptive statistics were generated to provide basic sample characteristics such as socio-demographic characteristics. Secondly, bivariate logistic regression of the outcome variable, modern contraceptive use, and explanatory variables was carried out to determine significance of associations between the outcome variable and explanatory variables. Explanatory variables were considered significant at a p-value of 0.05 or less. Multivariate logistic regression was fitted to predict correlates of contraceptive use in the presence of explanatory variables. All the analyses were weighted to account for differences in sampling probabilities. We fit three models to assess the influence of migration stream as a key explanatory variable. Model I assesses the influence of migration stream and modern contraceptive use, model II adjusts for the influence of migration stream and socio-demographic factors and model III determines the influence of migration stream in the presence of both socio-demographic and socio-economic factors.

## Results

### Sample description

A description of the 5,905 women who use modern contraceptives is shown in Table 1. Slightly more than a third (34.5 %) of the respondents reported current use of a modern method of contraception. The use of modern contraceptives was high among non-migrant urban women 46.6 %, followed by rural-urban and urban-rural migrants at 44.4 and 38.5 % respectively. Considering age of the woman, modern contraceptive use was 44.0 % and 26.5 % among women aged 25–34 years and 15–24 years respectively. Women currently in marriage were the majority (41.4 %) of modern contraceptive users. Similarly, a vast majority of women with 3–5 children (42.2 %) and protestantants (36.5 %) reported using modern contraceptives. The use of modern contraceptives was also high among women in Central (52.5 %), Nairobi (42.7 %) and Eastern (38.8 %) regions. A large proportion of women with secondary/higher education (42.4 %), from medium (38.8 %) and high (42.7 %) income households and those who engaged in professional work (42.5 %) reported use of modern contraceptives. Access media messages on family planning also contributed to the use of modern contraceptives (39.6 %).

**Table 1:** Percent distribution of socio-demographic and socio-economic characteristics of migrant and non-migrant women using modern contraceptives in Kenya

Characteristics	Percent (%)	95 % CI	N [Weighted]
Migration stream			
Non-migrant urban	46.6	[37.1–56.4]	263
Non-migrant rural	23.7	[20.5–27.3]	1,430
Urban-urban	38.3	[31.0–46.2]	644
Urban-rural	38.5	[33.6–43.6]	585
Rural-urban	44.4	[39.7–49.2]	620
Rural-rural	35.1	[32.3–37.9]	2,361
Socio-demographic factors			
Age			
15–24	26.5	[23.4–29.8]	1,759
25–34	44.0	[40.9–47.1]	2,280
35–49	30.5	[27.3–34.0]	1,866
Marital status			
Never married	17.3	[14.5–20.6]	1,123
Currently married	41.4	[38.8–44.1]	4,035
Formerly married	23.0	[19.0–27.6]	746
Living children			
None	16.4	[13.0–20.5]	785
1–2	38.6	[35.5–41.7]	2,215
3–5′	42.2	[39.0–45.4]	2,084
6+	21.4	[17.8–25.5]	820
Religion			
Catholic	35.7	[31.7–39.9]	1,287
Protestant	36.5	[34.3–38.8]	4,043
Muslim/other	17.8	[12.7–24.4]	576
Fertility preference			
Want another child	29.9	[27.5–32.6]	2,817
Undecided	33.6	[22.9–46.4]	177
Want no more	39.0	[36.3–41.8]	2,911
Region			
Nairobi	42.7	[37.2–48.3]	527
Central	52.5	[47.0–58.0]	626
Coast	30.9	[23.8–39.0]	474
Eastern	38.8	[33.7–44.3]	981
Nyanza	28.8	[25.6–32.3]	990
Rift valley	29.2	[25.0–33.8]	1,632
Western	33.9	[30.2–37.8]	561
North Eastern	3.9	[1.3–11.0]	114
Marital duration			
Never married	17.3	[14.5–20.6]	1123
0–9 years	42.6	[39.2–46.1]	2095
10–24 years	41.1	[37.8–44.6]	1621
20 and more years	26.6	[23.1–30.4]	1067
Socio-economic factors			
Education			
None	10.1	[7.0–14.3]	576
Primary	34.0	[31.5–36.6]	3327
Secondary/Higher	42.4	[39.2–45.7]	2002
Wealth index			
Low	21.3	[18.6–24.3]	1881
Medium	38.8	[35.6–42.2]	2132
High	42.7	[40.1–45.4]	1892
Occupation			
Not working	26.7	[24.1–29.4]	1997
Professional/technical/manager/clerical/sales/service	42.5	[39.5–45.5]	1805
Agri-employee/household domestic/manual	35.1	[32.2–38.2]	2103
Heard FP on media			
No	19.8	[17.5–22.4]	1530
Yes	39.6	[37.6–41.7]	4375
Total (N)	34.5	[32.6–36.5]	5905

*CI* confidence interval

### Correlates of modern contraceptive use

Regression models were fit to identify correlates of modern contraceptive use with the key explanatory variable, migration stream. We identified socio-demographic and socio-economic factors such as migration stream, age, marital status, number of living children, religion, fertility preference, region of residence, marital duration, level of education, wealth index, occupation and access to media messages on family planning, as shown in Table 2 to be significantly (*p* < 0.001) associated with modern contraceptive use. Multivariate logistic regression adjusted for various factors in Model I-III where most of the associations remained significant as shown in Table 3. The reference category for each variable is given in parentheses. Modern contraceptive use was significantly higher among women in all migration streams (non-migrant urban (OR = 2.8, *p* < 0.001), urban-urban (OR = 2.0, *p* < 0.001), urban-rural (OR = 2.0, *p* < 0.001), rural-urban (OR = 2.6, *p* < 0.001), rural-rural (OR = 1.7, *p* < 0.001), than non-migrant rural women as shown in model I.

**Table 2:** Association between modern contraceptive use and background characteristics of migrant and non-migrant women 15–49 years in Kenya

Characteristics	Odds ratio	95 % CI
Migration stream [Non-migrant rural]		
Non-migrant urban	2.807***	[1.82–4.33]
Urban-urban	1.994***	[1.37–2.91]
Urban-rural	2.009***	[1.52–2.66]
Rural–urban	2.563***	[1.96–3.35]
Rural-rural	1.734***	[1.43–2.11]
Socio-demographic factors		
Age [15–24 years]		
25–34	2.176***	[1.76–2.69]
35–54	1.219	[0.99–1.51]
Marital status [Formerly married]		
Never married	0.700*	[0.51–0.97]
Currently married	2.365***	[1.84–3.03]
Living children [None]		
1–2	3.194***	[2.38–4.28]
3–5′	3.711***	[2.72–5.06]
6+	1.389	[0.98–1.96]
Religion [Catholic]		
Protestant	1.037	[0.85–1.26]
Muslim/Other	0.391***	[0.26–0.59]
Fertility preference [Want another child]		
Undecided	1.186	[0.70–2.01]
Want no more	1.495***	[1.27–1.76]
Region [Nairobi]		
Central	1.485*	[1.08–2.04]
Coast	0.601*	[0.39–0.92]
Eastern	0.852	[0.62–1.17]
Nyanza	0.544***	[0.41–0.72]
Rift valley	0.553***	[0.40–0.76]
Western	0.689**	[0.52–0.91]
North Eastern	0.055***	[0.02–0.17]
Marital duration [20 and more years]		
Never married	0.579***	[0.44–0.76]
0–9 years	2.055***	[1.64–2.58]
10–24 years	1.932***	[1.56–2.39]
Socio-economic factors		
Education [None]		
Primary	4.601***	[3.02–7.01]
Secondary/Higher	6.588***	[4.24–10.25]
Wealth index [Low]		
Medium	2.345***	[1.84–2.99]
High	2.759***	[2.26–3.37]
Occupation [Not working]		
Professional/technical/manager/clerical/sales/service	2.027***	[1.72–2.40]
Agri-employee/household domestic/manual	1.485***	[1.24–1.77]
Heard FP on media [No]		
Yes	2.658***	[2.25–3.14]

*p < 0.05; **p < 0.01; ***p < 0.001; *CI* - Confidence interval - 95 %

**Table 3:** Odds ratio of modern contraceptive use among migrant and non-migrant woman in Kenya

	Model I		Model II		Model III	
Characteristics	Odds ratio	95 % CI	Odds ratio	95 % CI	Odds ratio	95 % CI
Migration stream [Non-migrant rural]						
Non-migrant urban	2.807***	[1.82–4.33]	3.380***	[1.93–5.92]	2.137**	[1.20–3.80]
Urban-urban	1.994***	[1.37–2.91]	1.885***	[1.23–2.88]	1.120	[0.69–1.81]
Urban-rural	2.009***	[1.52–2.66]	1.520**	[1.14–2.03]	1.208	[0.88–1.65]
Rural–urban	2.563***	[1.96–3.35]	2.054***	[1.53–2.77]	1.373	[0.97–1.94]
Rural-rural	1.734***	[1.43–2.11]	1.181	[0.96–1.46]	1.128	[0.91–1.40]
Socio-demographic factors						
Age [15–24 years]						
25–34			1.362**	[1.07–1.74]	1.148	[0.88–1.49]
35–54			0.924	[0.67–1.27]	0.704*	[0.51–0.97]
Marital status [Formerly married]						
Never married			1.411	[0.94–2.13]	1.256	[0.83–1.89]
Currently married			2.708***	[2.08–3.53]	2.631***	[2.03–3.41]
Living children [None]						
1–2			1.750***	[1.27–2.42]	1.808***	[1.31–2.50]
3–5′			1.822***	[1.25–2.65]	2.184***	[1.50–3.19]
6+			0.938	[0.61–1.43]	1.347	[0.88–2.06]
Religion [Catholic]						
Protestant			1.054	[0.85–1.30]	1.009	[0.81–1.25]
Muslim/Other			0.427***	[0.28–0.65]	0.610*	[0.39–0.94]
Fertility preference [Want another child]						
Undecided			1.145	[0.64–2.04]	1.142	[0.65–2.01]
Want no more			1.547***	[1.25–1.92]	1.496***	[1.21–1.85]
Region [Nairobi]						
Central			2.111***	[1.45–3.08]	1.586**	[1.09–2.31]
Coast			0.891	[0.64–1.25]	0.977	[0.70–1.36]
Eastern			1.249	[0.84–1.85]	1.228	[0.84–1.80]
Nyanza			0.768	[0.55–1.08]	0.678*	[0.48–0.96]
Rift valley			0.828	[0.60–1.14]	0.794	[0.58–1.09]
Western			1.014	[071–1.44]	0.959	[0.69–1.34]
North Eastern			0.172***	[0.06–0.52]	0.351*	[0.13–0.97]
Marital duration [20 and more years]						
Never married			1.000	-	1.000	-
0–9 years			1.551**	[1.11–2.16]	1.519*	[1.08–2.13]
10–24 years			1.513***	[1.17–1.96]	1.452**	[1.11–1.90]
Socio-economic factors						
Education [None]						
Primary					2.031***	[1.34–3.07]
Secondary/Higher					2.620***	[1.66–4.13]
Wealth index [Low]						
Medium					1.631***	[1.26–2.11]
High					1.623**	[1.17–2.26]
Occupation [Not working]						
Professional/technical/manager/clerical/sales/service					1.525***	[1.22–1.91]
Agri-employee/household domestic/manual					1.304**	[1.08–1.58]
Heard FP on media [No]						
Yes					1.838***	[1.50–2.25]

*p < 0.05; **p < 0.01; ***p < 0.001; *CI* - Confidence interval - 95 %

In model II, we adjust for the effects of migration stream and socio-demographic factors and observe an increased likelihood to use modern contraceptives among women in all migration streams and particularly non-migrant urban women, (OR = 3.4, *p* < 0.001) compared to non-migrant rural women. Women aged 25–34 years were also 1.3 times (*p* < 0.05) more likely to use modern contraceptives than those below 25 years. Similarly, there was a higher likelihood to use modern contraceptives among currently married women (2.7 times, *p* < 0.001) compared to formerly married women. Having 1–2 children (1.8 times, *p* < 0.001) and 3–5 children (1.8 times, *p* < 0.001) increased the likelihood to use modern contraceptives compared to having no children. Women from the Muslim or other faiths were less likely (0.6 times, *p* < 0.001) to use modern contraceptives than women who subscribe to the Catholic faith. There was an increased likelihood to use modern contraceptives among women who did not desire more children (1.5, *p* < 0.001) compared to their counterparts who wanted another child. Women from Central region (2.1 times, *p* < 0.001) were more likely to use modern contraceptives than those from Nairobi, while those from North Eastern were less likely (0.8 times, *p* < 0.001) to use modern contraception compared to those from Nairobi.

In model III, we adjust for the effects of migration stream, socio-demographic and socio-economic factors, and according to the results migration stream remains an important factor in determining modern contraceptive use where non-migrant urban women (2.1 times, *p* < 0.01) were more likely to use modern contraceptives than non-migrant rural women. Older women (35 years and above) were less likely to use modern contraceptives (*p* < 0.05) than their younger counterparts (under 25 years). There was an increased likelihood to use modern contraceptives for women with 1–2 children (1.8 times, *p* < 0.001), 3–5 children (2.2 times, *p* < 0.001) compared to those with no children. Similarly, women who did not desire to have more children (1.5 times, *p* < 0.001) were more likely to use modern contraceptives than those wanting another child. Residents of Central region were more likely to use modern contraceptives, than those residing in Nairobi, on the hand, women from Nyanza and North Eastern region were less likely to use modern contraceptives compared to those from Nairobi region.

Having at least primary education (*p* < 0.001) increased the likelihood to use modern contraceptives twofold while women engaged in professional and agriculture/household related occupations were 1.5 times (*p* < 0.001) and 1.3 times (*p* < 0.01) respectively more likely to use modern contraceptives that those not engaged in any economic activity. Socio-economic status was also associated with modern contraceptive use, women from at least medium wealth households were more likely (*p* < 0.001) to use modern contraceptives than those from low wealth households. Exposure to family planning messages from the media increased the likelihood to use modern contraceptives 1.8 times, *p* < 0.001 compared to non-exposure to media messages.

## Discussion

This study is an attempt to explore the effect of migration on modern contraceptive use among women aged 15–49 years in Kenya. The bivariate and multivariate logistic analysis results indicated that migration stream was significantly associated with current use of modern contraceptive methods. The analysis shows that, migrant women, regardless of their migration stream, have a higher likelihood to use modern contraceptives than non-migrant rural women. Despite this finding, our results further show that non-migrant urban women were more likely to use modern contraception than women from different migration streams a possible indication of the adaption effect. Generally, contraceptive use is higher in urban than rural areas, thereby giving the non-migrant urban women advantages over women from all other migration streams [16, 21]. On the other hand, rural-urban migrants were more likely to use modern contraceptives compared to their non-migrant rural counterparts, possibly an indication of the adaption effect that assumes that as these women move to urban areas, they acquire urban characteristics including adoption of contraceptive use in the process of acquiring and adapting to the way of the urban area [22, 23]. Brockerhoff in a 1995 study also found migrant women to change their characteristics and adapt those of their destination including fertility behaviour [15]. The pattern of modern contraceptive use depicted among migrant women in this study is typical of the configuration of contraceptive services in Kenya where greater access is reported in urban than rural areas [14].

Migration streams is significantly but diversely related to contraceptive use in Kenya as also shown elsewhere in sub-Saharan Africa [17]. Rural-urban migrants and urban-urban migrants have a higher likelihood of using contraceptives than rural-rural migrants and non-migrant rural women. Among non-migrants, place of residence (mostly urban residence) has been found to be a determining factor in modern contraceptives use. However, migration occurring between various locations, especially from rural to urban areas, seems to result in greater changes in contraceptive attitudes and behaviours usually attributed to external stimuli [7]. Internal migration (migration within a country) and to some extent external migration (migration to a different county or continent) is most often associated with social, cultural, economic and environmental changes which can spur attitude and behaviour change [2]. More so, the fact that migrant women are more likely to use modern contraceptives supports the notion that innovative ideas or information on fertility regulation are more reinforced in urban than rural settings which are less developed, and traditional. Furthermore, this analysis is supportive of the hypothesis that exposure to urban environments is associated with better socio-economic indicators and in essence validates the self-selection theory [1].

While adjusting for the effects of socio-demographic characteristics of the migrant and non-migrant women, it is apparent that migration status remains important in determining modern contraceptive use. Additionally, women in their prime reproductive ages, 25–34 years, being currently married, having 1–5 children, desiring no more children and being resident of Central region had increased odds of modern contraceptive use. These can be attributed to the disruption effect that seems incompatible with childbearing. The disruption effect comes into play by delaying childbearing either by separation or time taken to adjust at the new environment [24]. When we adjust for the effects of both socio-demographic and socio-economic factors, the effect of migration reduces possibly confirming the selection effect which states that migrants are self-selecting group with characteristics that cause delays in childbearing [15]. Lending further credence to the socio-economic differences in rural and urban areas in Kenya, where higher values of economic indicators are observed for the urban areas and less so for most rural areas. In the multivariate analysis, migration status, age of women, number of living children, education, occupation, being currently married, listenership to family planning messages on media, region of residence and wealth index were seen to play a vital moderating role in the use of modern contraceptive methods. These findings are consistent with other studies conducted in sub-Saharan Africa [25, 26]. More so, although socio-economic differentials in knowledge and perceptions, access to health facilities and health information, and associated costs influence uptake of modern contraceptive use among migrants, the specific mitigating factors warrants further study.

The main strength of this study is the use of national population-based data hence the findings can be generalized at the country level. However, the major limitation is the use of one data point which fails to document the occurrence of multiple events over time. This could have led to potential misclassification of contraceptive use or migration status, however, with regards to the latter, most migrants in this study reported that they had resided in the current place of residence for a duration of time, and therefore the effects of misclassification on contraceptive use are likely minimal. This study indicates the need for further research on migrants and non-migrants, to clarify the role of rural-urban environments and individual behaviors in promoting modern contraceptive use as well as interventions to promote family planning methods in rural areas.

## Conclusion

This study confirms the central role played by migration notably migration streams which have a direct consequence on individual’s social, cultural, economic and environmental changes which in effect impacts contraceptive use. The evidence from this study can be useful to policy makers, programme implementers and stakeholders to help inform future interventions and also improve health services among various categories of the population in Kenya. Programmatically, the differentials in modern contraceptive use by the different migration streams should be considered when designing programmes in response to family planning needs of migrant and non-migrant women. It was also evident that migrants exhibited higher modern contraceptive use due to access to higher levels of education, employment among other factors. Enabling access to such services by the government will help increase higher contraceptive use especially in rural areas where the provision of similar services remains inadequate.

## References

[1] Mishra P, Mishra B, Dixit SJ (2014). Internal migration and current use of modern contraception methods among currently married women age group between (15–49) years in India.

[2] NCPD. Kenya Population Situation Analysis. Nairobi, Kenya: Government of Kenya and UNFPA Kenya Country Office; 2013.

[3] Aworemi JR, Abdul-Azeez IA, Opoola NA. An appraisal of the factors influencing rural-urban migration in some selected local government areas of Lagos State Nigeria. J Sustain Dev. 2011;4(3):136.

[4] International Organization for Migration (IOM) (2009). Gender and labour migration in Asia.

[5] Martin Forbes S (2003). Women and Migration, in Consultative Meeting on “Migration and Mobility and how this movement affects Women”.

[6] Keil, K. The Female Face of Migration: Advocacy and best practices for women who migrate and the families they leave behind. Caritas Intarnationalis. 2012. http://www.caritas.org/includes/pdf/advocacy/FFMCaritasPolicyDoc.pdf.

[7] Omondi OC, Ayiemba EHO. Fertility differentials in Kenya: the effect of female migration. Afr Popul Stud. 2005;20(2):25–42.

[8] de Plessis G (1995). The development of demography in South Africa: a relevant social science or a tool for counting subgroups?. S Afr J Demography.

[9] World Health Organization (WHO) (2007). Adolescent Migrants in the Greater Mekong Sub-region: Are they equipped to protect themselves against sexual and reproductive health risks?.

[10] Kessler K, Quezada L, Goldenberg S (2010). Contraceptive Use in a Community of International Migration.

[11] Singh Kant V (2012). Impact of residential status of women on fertility. Aust J Sci Res.

[12] Goldstein S (1978). Migration and fertility in Thailand, 1960–1970. Can Stud Popul.

[13] Martine G, Alves Eustaquio J, Cavenaghi S (2013). Urbanization and Fertility Decline: Cashing in on Structural Change.

[14] Kenya National Bureau of Statistics (KNBS) and ICF Macro (2010). Kenya Demographic and Health Survey 2008–09.

[15] Brockerhoff M (1995). Fertility and family planning in African cities: the impact of female migration. J Biol Sci.

[16] Brockerhoff M, Biddlecom AE (1999). Migration, sexual behavior and the risk of HIV in Kenya. Int Migr Rev.

[17] Omondi Ochola C, Omondi Ochola C, Ayiemba EHO (1998). Contraceptive use dynamics among migrant women in Kenya. Afr Popul Stud.

[18] Moreno L. Residential Mobility and ContraceptiveUse in Northeastern Brazil, in DHS Working papers No.9. Macro International; 1994.

[19] Ojakaa D. The fertility transition in Kenya: patterns and determinants, in Department of Demography. University of Montreal. Canada; 2008.

[20] Ngom P, et al. Family planning needs in the context of the HIV/AIDS epidemic: Findings from a three-country assessment covering Kenya, South Africa and Zimbabwe, in XXV IUSSP International Conference. 2005: Tours, France.

[21] Kessler K, Goldenberg MS, Quezada L. Contraceptive use, unmet need for contraception, and unintended pregnancy in a context of Mexico-U.S. Migration. Migr Health, 2010. 2010. Field Actions Science Reports [Online], Special Issue 2. http://factsreports.revues.org/534.

[22] McKinney Barbara J (1993). Impact of rural-urban migration on migrant fertility in Senegal.

[23] Hendershot G (1976). Social Class, Migration, and Fertility in the Philippines. The Dynamics of Migration: Internal Migration and Migration and Fertility, in Interdisciplinary Communications Program, Occasional Monograph Series.

[24] Omoyeni TS (2013). Migration and Family Formation Dynamics in Nigeria: An Exploration of Linkages between Migration and Reproductive Behaviour.

[25] Lindstrom PD, Herrera Hernández C (2006). Internal migration and contraceptive knowledge and use in Guatemala. Int Fam Plan Perspect.

[26] Subariya L (2007). Internal Migration and the Use of Reproductive and Child Health Services in Peru, Demographic Health Survey.

